# Surface-enhanced Raman scattering of suspended monolayer graphene

**DOI:** 10.1186/1556-276X-8-480

**Published:** 2013-11-15

**Authors:** Cheng-Wen Huang, Bing-Jie Lin, Hsing-Ying Lin, Chen-Han Huang, Fu-Yu Shih, Wei-Hua Wang, Chih-Yi Liu, Hsiang-Chen Chui

**Affiliations:** 1 Department of Photonics, National Cheng Kung University, Tainan 70101, Taiwan; 2 Center for Nano Bio-Detection, National Chung Cheng University, Chiayi 621, Taiwan; 3 Institute of Atomic and Molecular Sciences, Academia Sinica, Taipei 10617, Taiwan; 4 Advanced Optoelectronic Technology Center, National Cheng Kung University, Tainan 70101, Taiwan

**Keywords:** Suspended graphene, Raman spectroscopy, SERS

## Abstract

**Abstract:**

The interactions between phonons and electrons induced by the dopants or the substrate of graphene in spectroscopic investigation reveal a rich source of interesting physics. Raman spectra and surface-enhanced Raman spectra of supported and suspended monolayer graphenes were measured and analyzed systemically with different approaches. The weak Raman signals are greatly enhanced by the ability of surface-enhanced Raman spectroscopy which has attracted considerable interests. The technique is regarded as wonderful and useful tool, but the dopants that are produced by depositing metallic nanoparticles may affect the electron scattering processes of graphene. Therefore, the doping and substrate influences on graphene are also important issues to be investigated. In this work, the peak positions of G peak and 2D peak, the *I*_2D_/*I*_G_ ratios, and enhancements of G and 2D bands with suspended and supported graphene flakes were measured and analyzed. The peak shifts of G and 2D bands between the Raman and SERS signals demonstrate the doping effect induced by silver nanoparticles by n-doping. The *I*_2D_/*I*_G_ ratio can provide a more sensitive method to carry out the doping effect on the graphene surface than the peak shifts of G and 2D bands. The enhancements of 2D band of suspended and supported graphenes reached 138, and those of G band reached at least 169. Their good enhancements are helpful to measure the optical properties of graphene. The different substrates that covered the graphene surface with doping effect are more sensitive to the enhancements of G band with respect to 2D band. It provides us a new method to distinguish the substrate and doping effect on graphene.

**PACS:**

78.67.Wj (optical properties of graphene); 74.25.nd (Raman and optical spectroscopy); 63.22.Rc (phonons in graphene)

## Background

Graphene, the thinnest *sp*^2^ allotrope of carbon arranged in a honeycomb lattice, has attracted many attentions because its unique and novel electrical and optical properties [1-3]. The wonderful and remarkable carrier transport properties of suspended graphene compared with supported graphene have been studied [4-9]. The performances of dopants, the effects of defects in graphene, and the phonon modes of suspended and supported graphenes vary but can be well understood using Raman spectroscopy [10-12]. Raman spectroscopy and surface-enhanced Raman spectroscopy (SERS) have been extensively applied to understand the vibration properties of materials [13-18], and they are regarded as powerful techniques in characterizing the band structure and detail of phonon graphene interaction [19-24]. The ability of SERS, a wonderful and useful technique used to enhance weak Raman signals, has attracted considerable attention. In previous SERS measurements, however, the doping induced by metallic nanoparticles on graphene deposition may affect the electron scattering processes of graphene. Otherwise, the metallic nanoparticles on graphene are also used as an electrode in graphene-based electronic devices [25,26]. Therefore, the effect of charged dopants and the substrate which affected graphene are both important issues to be investigated. In this work, the supported and suspended monolayer graphene samples were fabricated by micromechanical cleavage method. They were identified as the monolayer graphene by the optical microscopy and Raman spectroscopy because of the color contrast, various bandwidth, and peak position of 2D band with different graphene layers [27,28]. The Raman and SERS signals of suspended and supported graphenes can be measured and analyzed systematically. The peak positions of G and 2D bands, the *I*_2D_/*I*_G_ ratio, and enhancements of G and 2D bands were obtained, respectively. With our analysis, details about the effects of charged impurities and substrate can be realized. The peak shift of G and 2D bands and the *I*_2D_/*I*_G_ ratio are useful to demonstrate the dopants and substrate effects on the graphene. The well-enhanced G and 2D bands are obtained to enhance the weak Raman signals. Moreover, the enhancements of G band with respect to 2D band are found to be more sensitive to various substrate influences on the graphene surface. This paper provides a new approach to investigate the substrate and doping effect on graphene.

## Methods

**Figure 1:**
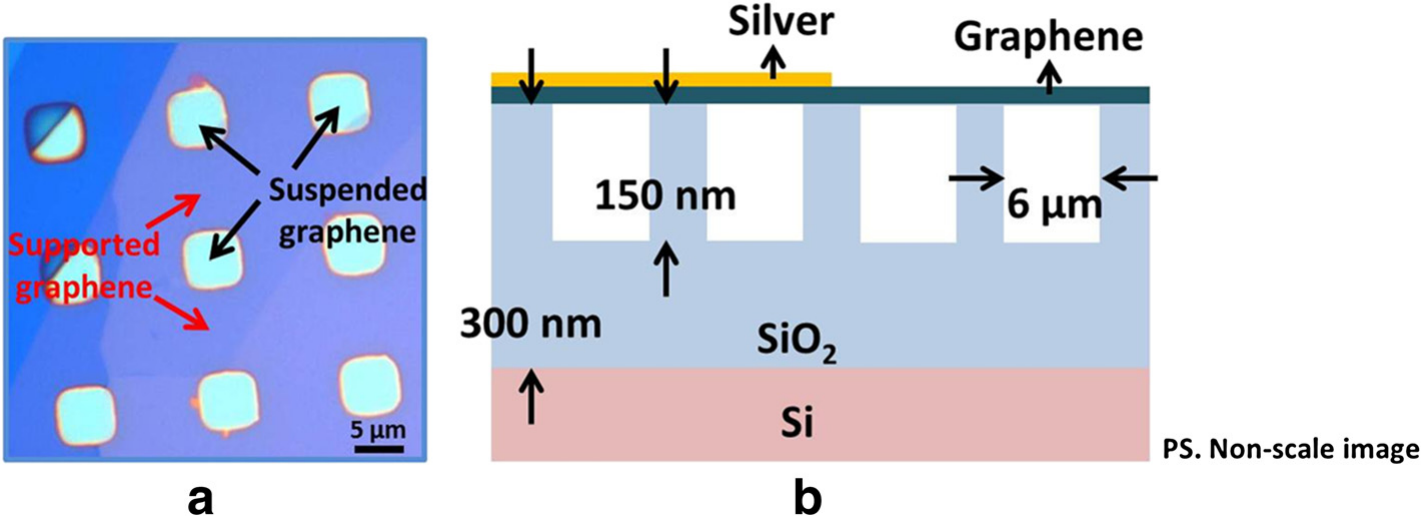
**Optical image of suspended and supported graphenes and their coverage by silver nanoparticles.** Optical image of suspended and supported graphenes **(a)** and their illustrations covered by silver nanoparticles **(b)**.

Suspended graphene was fabricated by mechanical exfoliation of graphene flakes onto an oxidized silicon wafer. The optical image of suspended and supported graphenes and the illustration of their coverage by silver nanoparticles are shown in Figure 1. Orderly arranged squares with areas of 6 μm^2^ were first defined by photolithography on an oxidized silicon wafer with an oxide thickness of 300 nm. Reactive ion etching was then used to etch the squares to a depth of 150 nm. Highly ordered pyrolytic graphite was consequently cleaved with the protection of scotch tape to enable the suspended graphene flakes to be deposited over the indents. To study the SERS, silver nanoparticles were deposited on the graphene flake at a deposition rate of 0.5 nm/min by a thermal deposition system. A 5-nm-thick layer of silver nanoparticles on the graphene flake was thus formed. To measure the graphene flake, a micro-Raman microscope (Jobin Yvon iHR550; HORIBA, Ltd., Minami-ku, Kyoto, Japan) was utilized to obtain the Raman and SERS signals of monolayer graphene. The monolayer graphene was identified through optical observation with various color contrast and by Raman spectroscopy with the different shape bandwidths and peak positions of 2D band under different graphene layers. During spectroscopic measurement, a 632-nm He-Ne laser was used as the excitation source; the power was monitored and controlled under 0.5 mW to avoid the heating of the graphene surface.

## Results and discussion

**Figure 2:**
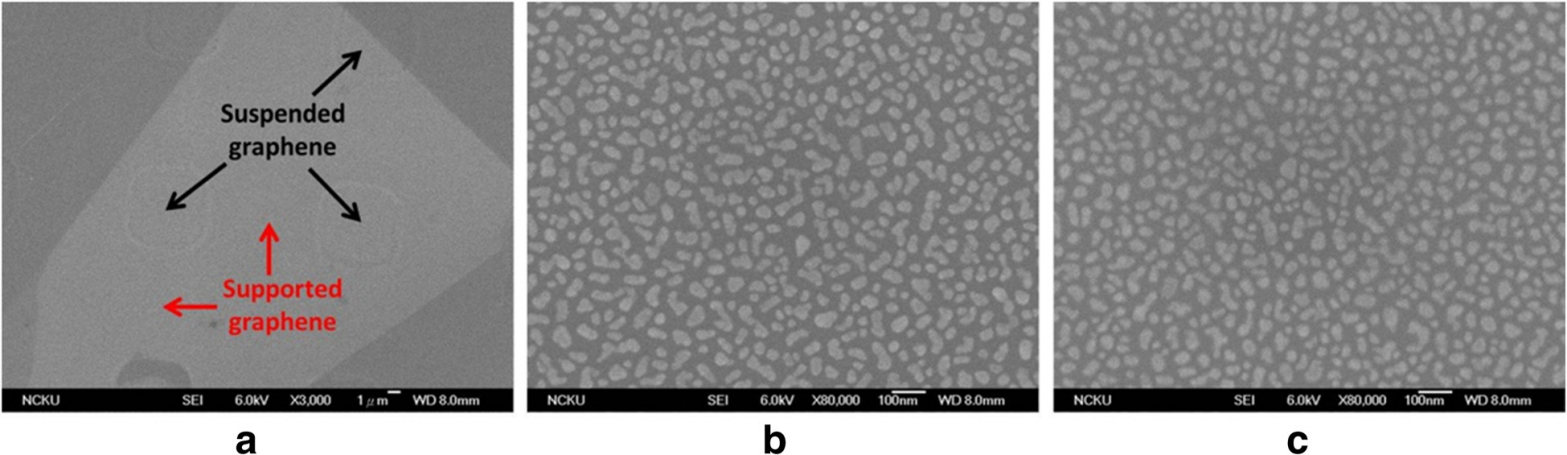
**SEM images. (a)** Supported and suspended graphenes which was identified as monolayer graphene. **(b)** Suspended graphene. **(c)** Supported graphene

To explore the SERS on graphene, the interactions between metallic nanoparticles and graphene surface has to be presumably understood. This is because the plasmonic resonances of nanoparticles with different shapes and sizes can affect the interactions between them, and then change the SERS signals [18,29-33]. Therefore, the morphologies of silver nanoparticles that covered the suspended and supported graphenes are to be investigated. The images of silver nanoparticles that covered suspended and supported graphenes were obtained by the scanning electron microscopy (SEM) and are shown in Figure 2a, b, c. The average size of silver nanoparticles were determined by the histogram analysis [34], of which the suspended graphene is 25.4 ± 2.2 nm and the supported graphene is 25.2 ± 2.4 nm. No clear size difference has been found between supported and suspended graphene flakes. In addition, their shapes are found in random form. It can also be seen that the silver nanoparticles deposited on the suspended and supported graphenes are in indistinguishable shape. Silver nanoparticles are therefore not contributing to any SERS variation.

**Figure 3:**
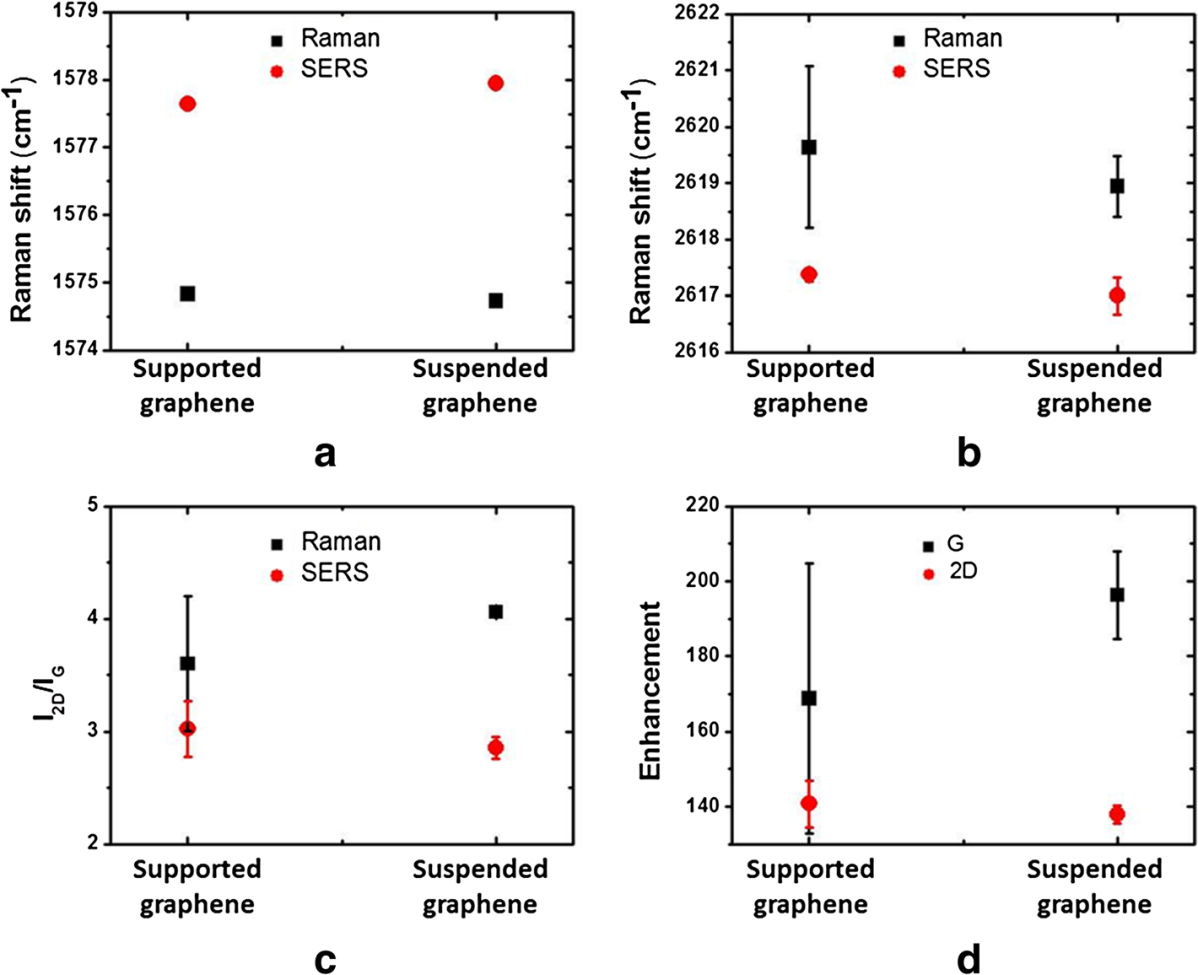
**Peak positions. (a)** G band and **(b)** 2D band of suspended and supported graphenes with Raman and SERS signals. **(c) ***I*_2D_/*I*_G_ ratios of suspended and supported graphenes with Raman and SERS signals. **(d)** Enhancements of G and 2D bands of suspended and supported graphenes.

According to previous work, the peak positions and *I*_2D_/*I*_G_ ratios of G and 2D bands were important indicators of doping effect on graphene [35-40], in which the *I*_2D_/*I*_G_ ratio is particularly more sensitive than the peak shifts to the doping effect. A lower *I*_2D_/*I*_G_ ratio is related to more charged impurities in graphene. The Raman and SERS signals of the suspended and the supported graphenes are shown in Figure 3a, b, c, d. The peak positions of G and 2D bands are presented in Figure 3a, b. Both the peak positions of G and 2D bands are indistinguishable between the suspended and supported graphenes, which reveals the difference in substrates which do not affect the graphene emission spectra. The G peak position of suspended and supported graphenes under Raman signals is both upshifted with respect to SERS signals, while the 2D peak under Raman signals is both downshifted with respect to SERS signals. According to previous work [35-37,39], the upshifting of G peak and the downshifting of 2D peak is caused by n-doping, as the silver nanoparticles were depositing on the graphene. The experimental results of this work have had a significant agreement with the previous research.

In order to minimize the random errors, each Raman spectra data point was obtained by five-time repetitions. As presented in Figure 3c, the *I*_2D_/*I*_G_ ratio of suspended graphene under Raman signals is 4.1 ± 0.1 and larger than supported graphene which is 3.6 ± 0.5, while the *I*_2D_/*I*_G_ ratio of suspended graphene on the SERS signals is around 2.9 ± 0.1 and smaller than supported graphene which is 3.0 ± 0.2. The result disclosed the substrate effect on the supported graphene is stronger than the suspended graphene. To our understanding, this above effect can be reasonable because SiO_2_/Si substrate has a direct contact on the graphene surface so the silver nanoparticles deposition causes stronger doping effect on the suspended graphene than supported graphene.

To understand the Raman and SERS signals, the enhancements of G and 2D bands with the suspended and supported graphenes are shown in Figure 3d, respectively. The enhancement is defined as the integrated intensities of SERS over Raman signals for the G and 2D bands, respectively. In our analysis, the enhancement of G band on supported graphene is 169.3 ± 20.1 and smaller than suspended graphene which is 196.2 ± 8.3, while the enhancement of 2D band with supported graphene which is 141.1 ± 4.3 is similar with suspended graphene which is 138.6 ± 1.6. The high enhancements of G and 2D bands are useful to enhance weak Raman signals, and the enhancements of G band with suspended and supported graphenes are both stronger than those of 2D band. Otherwise, the enhancement of G band is reduced obviously as silver nanoparticles deposited on suspended graphene, revealing that the enhancement of G band is sensitive to substrate effect on graphene with respect to 2D band. Based on the results, the doping effects with various substrates are obviously related to the enhancement of G band.

## Conclusions

In our work, Raman and SERS signals of supported and suspended monolayer graphenes were measured systematically. The peak positions of G and 2D bands and the *I*_2D_/*I*_G_ ratios were varied. The enhancement effect of suspended and supported graphenes was calculated and analyzed. The peak shifts of G and 2D bands and their Raman spectra and *I*_2D_/*I*_G_ of SERS signals are found very useful in the investigation of the substrate and doping effect on the optical properties of graphene. The enhancements of G and 2D bands have been found to cause the great improvement of weak Raman signals. Otherwise, the more sensitive enhancements of G band with respect to 2D band are related to the doping effect with various substrates that covered the graphene surface. The optical emission spectra of suspended and supported graphenes have provided us a with new identification approach to understand the substrate and doping effect on graphene.

## Competing interests

The authors declare that they have no competing interests.

## Authors' contributions

C-H and B-JL carried on the experimental parts: the acquisition of data and analysis and interpretation of data. C-H also had been involved in drafting the manuscript. H-YL and C-HH analyzed and interpreted the data. They also had been involved in revising the manuscript. F-YS and W-HW (Institute of Atomic and Molecular Sciences, Academia Sinica) prepared the samples, suspended graphene using by micromechanical method, and captured the OM and AFM images. C-YL have made substantial contributions to the conception and design of the study and revising it critically for important intellectual content. H-CC, the corresponding author, had made substantial contributions to the conception and design of the study and had been involved in drafting the manuscript and revised it critically for important intellectual content. All authors read and approved the final manuscript.
